# Renal and bone side effects of long-term use of entecavir, tenofovir disoproxil fumarate, and tenofovir alafenamide fumarate in patients with Hepatitis B: a network meta-analysis

**DOI:** 10.1186/s12876-023-03027-4

**Published:** 2023-11-10

**Authors:** Zekun Liu, Zhenzhen Zhao, Xuefeng Ma, Shousheng Liu, Yongning Xin

**Affiliations:** 1 Department of Infectious Disease, Qingdao Municipal Hospital, Qingdao University, Qingdao, 266071 Shandong China; 2 Clinical Research Center, Qingdao Municipal Hospital, Qingdao, 266071 Shandong China

**Keywords:** Entecavir, Tenofovir alafenamide, Tenofovir disoproxil fumarate, Hepatitis B virus, Chronic Hepatitis B

## Abstract

**Background:**

Nucleoside analogues are currently applied as a first-line treatment for chronic hepatitis B (CHB) patients. However, the long-term effects of this type of treatment on kidney and bone tissue need to be further investigated.

**Methods:**

We conducted a search of entecavir (ETV), tenofovir disoproxil fumarate (TDF), and tenofovir alafenamide fumarate (TAF) for treatment of CHB patients through October 29, 2023. Side effects of the three drugs were compared. Standardized mean difference (SMD), 95% confidence interval (95%CI), and surface under the cumulative ranking curve (SUCRA) were reported for each outcome. Further subgroup analysis was conducted according to duration of administration.

**Results:**

ETV and TAF exhibited less effect on estimated glomerular filtration rate (eGFR) than TDF (SMD = -3.60 (95%CI: -1.94 ~ -5.26) and SMD = -4.27 (95%CI: -2.62 ~ -5.93)). ETV also exhibited less effect on creatinine rise than TAF and TDF (SMD = -0.55 (95%CI: -0.09 ~ -1.01) and SMD = -0.61 (95%CI: -0.15 ~ -1.06)). Moreover, the effect of TAF on bone mineral density (BMD) was less than that of TDF (SMD = -0.02 (95%CI: -0.01 ~ -0.02)). The probabilities of the three drugs changing relevant indicators exhibited similar patterns: eGFR (TDF (100.0%) > ETV (41.2%) > TAF (8.8%)), creatinine (TDF (94.7%) > TAF (54.7%) > ETV (0.6%)), BMD (TDF (79.7%) > ETV (50.6%) > TAF (19.6%)), and blood phosphorus (TDF (90.6%) > TAF (49.8%) > ETV (9.7%)). After 6 and 24 months of treatment, no statistically significant difference in renal function or bone tissue was observed between ETV and TDF. However, greater adverse effects on renal function were observed for TDF than ETV at 60 months compared to 12 months. TDF also exhibited greater adverse effects on bone tissue than ETV at 36 months than at 12 months.

**Conclusions:**

Long-term administration of TDF has resulted in stronger adverse effects than TAF and ETV in regard to both renal function and bone tissue in CHB patients. The effect of TAF on creatinine increase was greater than ETV. The difference in side effects between ETV and TDF was independent of treatment duration.

**Supplementary Information:**

The online version contains supplementary material available at 10.1186/s12876-023-03027-4.

## Introduction

The most common chronic viral infection worldwide is caused by the hepatitis B virus (HBV). As a result, HBV is recognized as a major global public health threat and is currently the tenth leading cause of death worldwide. According to a 2019 authoritative review of hepatitis B, more than 257 million individuals worldwide are chronically infected with hepatitis B, and more than 887,000 individuals have had HBV infection as a cause of death. Therefore, the management and treatment of chronic hepatitis B is of great significance [1, 2].

Currently, there are two classes of antiviral drugs that are approved for the treatment of chronic hepatitis B virus infection: interferon alpha and nucleoside analogues. They act by continuously suppressing HBV replication and liver inflammation [3, 4]. Among the nucleoside analogues, entecavir (ETV), tenofovir disoproxil fumarate (TDF), and tenofovir alafenamide fumarate (TAF), are the most widely used as first-line treatments [5–9]. However, while nucleoside analogues have been shown to be safe and well tolerated, some patients experience cumulative toxicity after long-term use of oral antivirals, particularly bone and kidney damage [10–15]. These observations are consistent with nucleoside analogues being affected by renal metabolism, mainly in the proximal renal tubules, and this can lead to a decrease in estimated glomerular filtration rate (eGFR). Reduced phosphate reabsorption by proximal renal tubules can also lead to bone disease, with the main outcome being hypopuricemia. Alternatively, hypophosphatemia can lead to defects in bone mineralization, osteomalacia, and fractures. Considering that TAF exhibits high plasma stability, damage to kidneys and bones may differ [16–19].

To achieve anti-infectious activity, long-term, or even lifelong, treatment with nucleoside analogues is necessary. Therefore, in this study, we compared side effects associated with long-term use of nucleoside analogues on both renal function and bone tissue [10]. Previous studies and meta-analyses have identified side effects of ETV, TDF, and TAF. In a study that compared all three nucleoside analogues, the results were not statistically significant [12]. Furthermore, many of the studies published have only compared two of these drugs, and conclusions regarding side effects attributed to each of the three drugs were inconsistent. For example, Hou et al. reported that TDF exhibited greater side effects than TAF (*P* = 0.014) [20], while Iida-Ueno et al. reported that TDF exhibited greater side effects than ETV (*P* = 0.003) [21]. Meanwhile, Seto et al. demonstrated that TDF was associated with a greater number of side effects involving bone tissue compared to TAF (*P* < 0.001) [22]. Conversely, other studies have shown that pairwise comparisons between TDF, ETV, and TAF did not exhibit statistically significant differences [21, 23, 24].

A network meta-analysis can summarize data to make a sample size more sufficient, and can also combine both direct and indirect comparisons to draw an overall conclusion. As a result, a more accurate comparison of differences in side effects can be achieved. Therefore, we conducted a network meta-analysis to examine the safety of long-term administration of ETV, TAF, and TDF in regard to bone and kidney.

## Methods

### Research search and selection

**Fig. 1:**
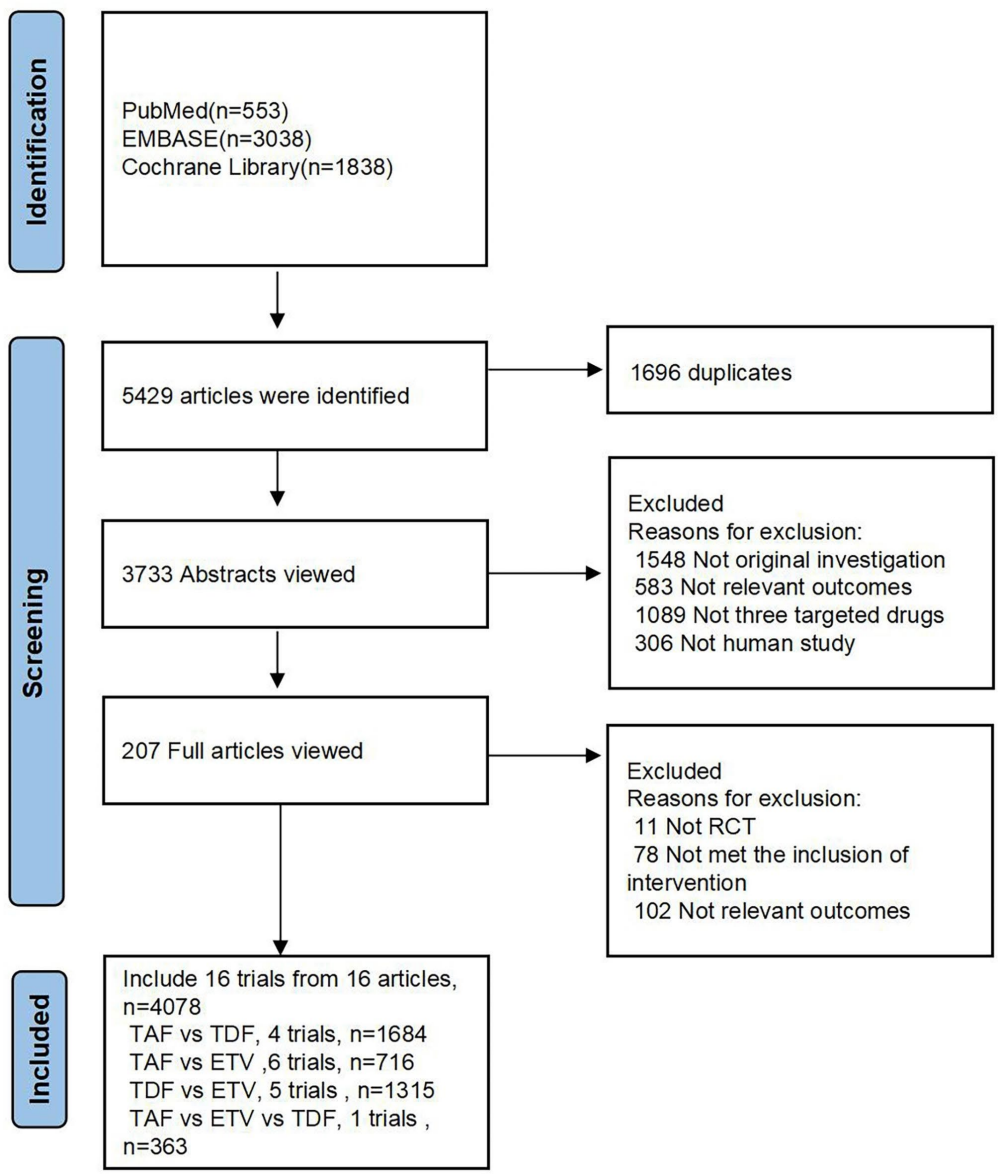
Overview of literature search and selection

Two researchers separately screened the PubMed, EMBASE, and Cochrane Library search engines for randomized controlled trials, hepatitis B, and all spellings of ETV, TDF, and TAF. References of the identified trials were also examined. After closely screening the full text content of the trials of interest, a final selection of articles was made (Fig. 1).

### Inclusion criteria

As of October 29, 2023, all randomized controlled trials involving patients with chronic hepatitis B treated with nucleoside analogues, ETV, TAF, and TDF were selected. Most of these trials provided comparative evaluations of the efficacy and side effects of these three nucleoside analogues in patients with chronic hepatitis B after a period of treatment. We used quantitative measures to assess damage to renal function and bone tissue. Among them, decrease in eGFR and increase in creatinine were used to indicate injury to renal function. Meanwhile, decreased bone mineral density and blood phosphorus were used as indications of bone injury.

### Exclusion criteria

Studies involving combination therapy, including interferon and nucleoside analogue combination therapy and multiple nucleoside analogue combination therapy, were excluded. In addition, studies that switched between treatments to another drug, studies in which therapeutic agents met the requirements yet the results were qualitative (e.g., renal impairment evaluated according to staging of chronic kidney disease), and studies that did not include renal function and bone examination indicators were excluded.

### Data extraction and evaluation

Information was extracted from each included article, including patient status, drug type, drug dosage, duration, and changes in creatinine, eGFR, blood phosphorus, and bone mineral density after ETV, TDF, or TAF treatment for various periods of time. Data quality was evaluated as data were extracted.

### Network geometry

Stata 15 software was used to draw a network geometry with three nodes representing ETV, TDF, and TAF drug treatments, respectively. Comparisons between the three drugs are represented by lines between the nodes.

### Risk assessment of bias

The bias risk assessment tool, Revman5.4, was used to assess bias risk and generate bias risk graphs for all of the selected studies.

### Summary measures

Both mean difference and standard deviation of continuous variables were examined in this study. To evaluate the effect of ETV, TDF, and TAF on renal function, changes in eGFR and serum creatinine levels before and after treatment were determined. The former was calculated by using a formula that includes serum creatinine values and other factors (e.g., age, gender, race), while the latter measures creatinine levels in blood [25]. This index fully corrects the influence of other factors on serum creatinine and can reflect the overall situation of the patient more comprehensively. To evaluate a possible effect on bone tissue, differences in bone mineral density and blood phosphorus concentration before and after medication were examined. In the physiological state, bone mineral density of human bone varies with age. In the pathological state, certain drugs can induce changes in bone mineral density. A decrease in blood phosphorus concentration can indicate osteoporosis. Greater sequential variation indicates greater effects of these drugs on renal function or bone. For a subset of studies which reported median and quartile spacing values, these values were converted to mean difference and standard deviation values using mathematical formulas. Some of these results are graphically represented and are based on the coordinates obtained.

### Analytical method

A random effects model was applied to compare drug outcomes. Direct comparisons between ETV, TDF, and TAF were analyzed to determine the magnitude of the adverse effects affecting renal function and bone. Following the network meta-analysis, a subgroup analysis was conducted according to the overall conclusions and data distribution. RevMan 5.4 software and Stata 15 software were used to analyze bias and perform data analysis, respectively.

## Results

### Study selection

After conducting a literature search, we selected sixteen randomized controlled trials involving 4278 adults with chronic hepatitis B who were treated with ETV, TDF, or TAF (Fig. 1). Data regarding changes in renal function and bone were examined.

### Study characteristics

One of the randomized controlled trials compared all three drugs, while the other fifteen trials only compared any two of the three drugs. The patients in all of the studies were from Asia, including China, South Korea, and Japan. Therefore, the findings represent a limited ethnicity pool. However, they do provide clinical evidence of the adverse effects of different drugs on renal function and bone. The basic characteristics of these studies are summarized in Table 1.

**Table 1:** Overview of study characteristics

Included studies (First author, year of publication)	Experimental group			Control group			Duration
	Sample size	Mean age (y)	Intervention	Sample size	Mean age (y)	Intervention	
Hu et al., 2020	678	59.4 ± 11.1	ETV	216	56.1 ± 11.6	TDF	5 years
Byun et al., 2021	87	56.4 ± 9.4	TAF	87	53.3 ± 9.5	TDF	48 weeks
Hou et al., 2021	227	38.00 ± 12.75	TAF	107	40.00 ± 13.25	TDF	144 weeks
Kim et al., 2020	37	55.8 ± 7.4	TDF	132	54.9 ± 7.1	ETV	3 years
Jeong et al., 2021	163	51.0 ± 12.6	ETV	154	51.2 ± 10.0	TDF	48 weeks
Jeong et al., 2021(2)	163	51.0 ± 12.6	ETV	46	51.00 ± 9.26	TAF	48 weeks
Ueno et al., 2019	19	56.0 ± 10.0	TDF	8	54.0 ± 10.0	ETV	48 weeks
Lee et al., 2020	102	54.0 ± 6.4	TDF	104	54.9 ± 7.8	ETV	60 months
Hagiwara et al., 2019	24	55.0 ± 12.0	ETV	24	61.0 ± 13.0	TAF	48 weeks
Hagiwara et al., 2021	32	54 ± 11	ETV	48	59.0 ± 12.0	TAF	96 weeks
Inada et al., 2021	66	68 ± 12	ETV	11	69.00 ± 7.25	TAF	24 weeks
Inoue et al., 2021	12	63.0 ± 15.5	TDF	7	48.0 ± 20.0	ETV	24 months
Itokawa et al., 2021	71	61.0 ± 12.5	TAF	71	58.0 ± 11.0	ETV	48 weeks
Kaneko et al., 2019	45	45.8 ± 14.0	TDF	14	47.9 ± 10.5	TAF	48 weeks
Uchida et al., 2020	92	62.0 ± 4.75	ETV	127	65.0 ± 4.75	TAF	48 weeks
Li et al., 2021	75	46.5 ± 12.4	TAF	75	48.7 ± 10.7	ETV	24 weeks
Seto et al., 2018	746	40.0 ± 11.8	TAF	371	41.0 ± 12.3	TDF	96 weeks

### Network structure

**Fig. 2:**
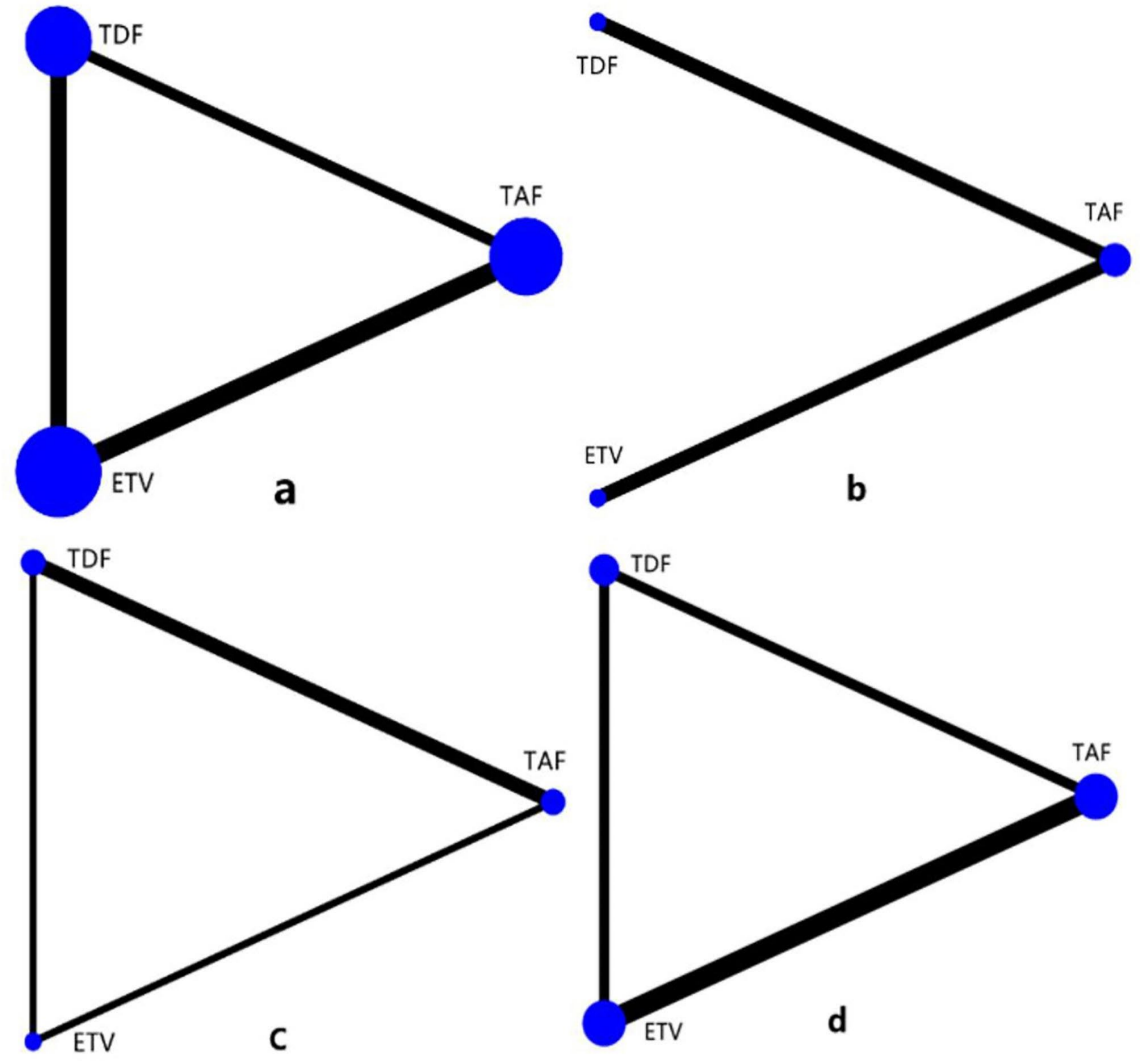
Geometry for the network meta-analysis. The size of the blue nodes is proportional to the number of patients using the drug, while the width of the black line is proportional to the number of studies comparing the two drugs. Figures a-d represent studies of eGFR, bone mineral density, creatinine, and blood phosphorus, respectively

**Fig. 3:**
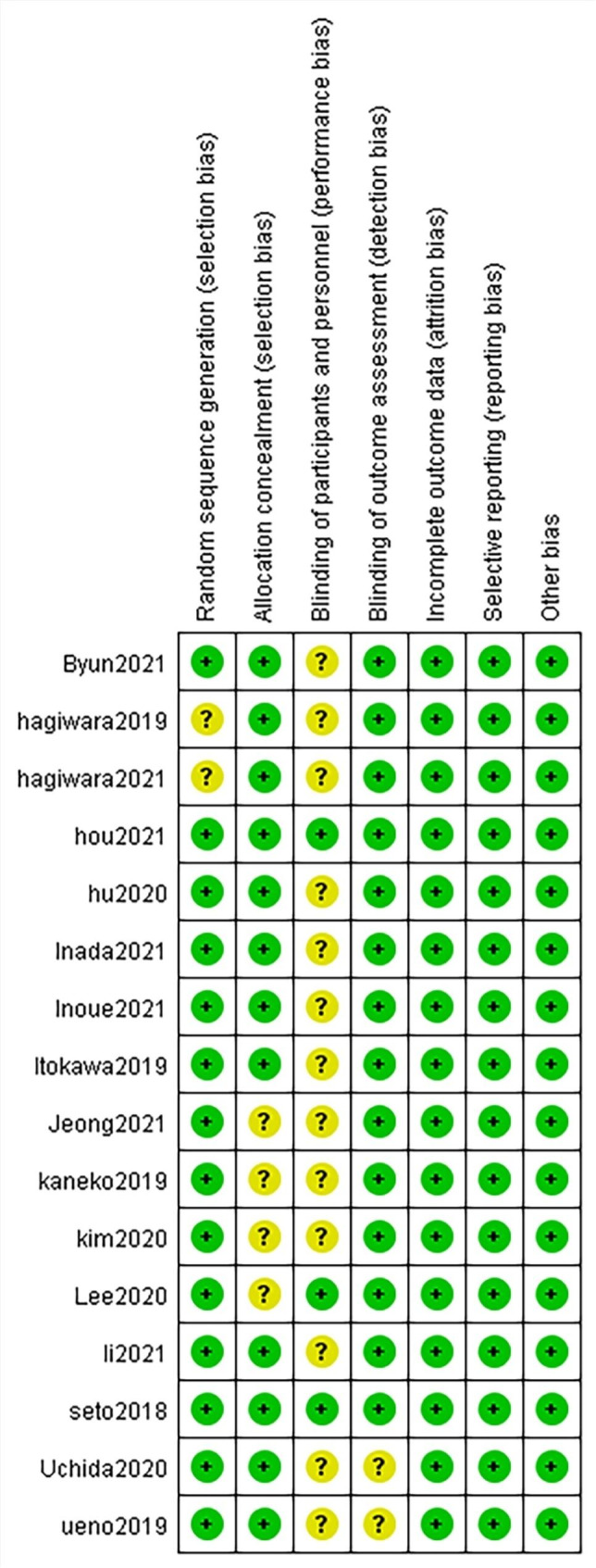
Risk of bias summary. Green indicates low risk, yellow indicates the risk is unclear, and red indicates high risk

In the network meta-analysis performed, renal function-related eGFR, creatinine, bone mineral density, and blood phosphorus concentration were examined. In the network geometric structure shown in Fig. 2, TAF, TDF, and ETV are represented as nodes, with corresponding comparisons shown as links between the nodes. The size of the blue nodes is proportional to the number of patients using the drug, while the width of the black line is proportional to the number of studies comparing two drugs. As shown in Fig. 3, the largest number of studies conducted to date are related to eGFR. Studies comparing the effects of TDF and ETV on BMD were not included, while studies comparing other indicators and drugs were included.

### Risk of bias

Two of the authors independently assessed all of the included studies using the Revman5.4 risk of bias tool (Fig. 3). The risk of bias was generally low, and all of the studies were included in the systematic review.

### Synthesis of results

#### Kidney events

**Fig. 4:**
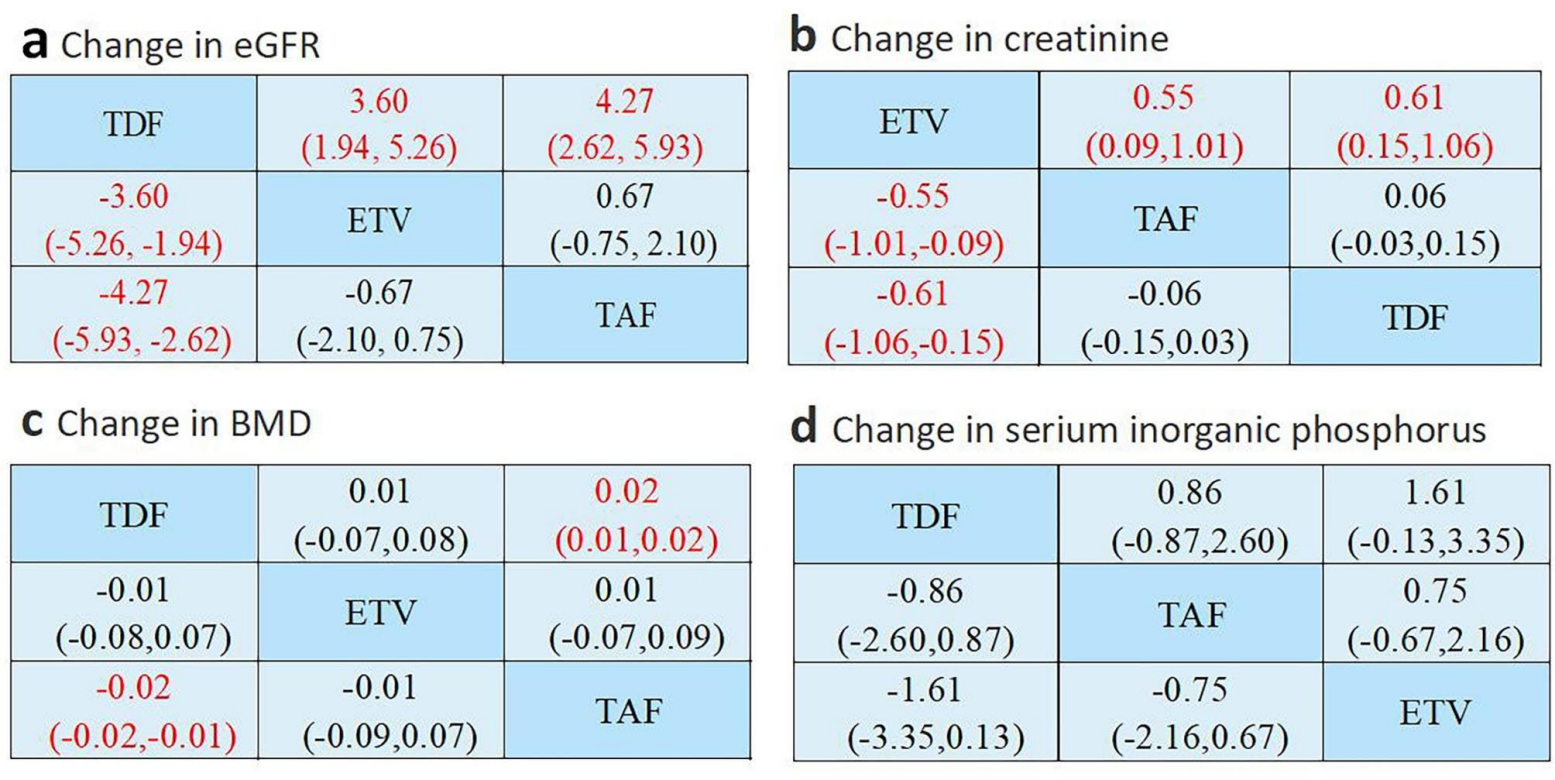
Network meta-analysis comparisons for side effect. Data are SMD (95% CI) in the column-defining treatment compared with the row-defining treatment. Taking SMD = 0 as the standard, negative value represents the strong decreasing effect or weak increasing effect on the index, while positive value represents the weak decreasing effect or strong increasing effect on the index. (a) changes in eGFR, (b) changes in creatinine, (c) changes in bone mineral density, and (d) changes in blood phosphorus

ETV and TAF exhibited less influence on eGFR reduction than TDF (SMD = -3.60; 95%CI: -1.94 ~ -5.26 and SMD = -4.27; 95%CI: -2.62 ~ -5.93, respectively). In contrast, the effect of ETV and TAF on eGFR reduction was not statistically significant (SMD = -0.67; 95%CI: -2.10 ~ 0.75) (Fig. 4a).

An analysis of the creatinine data showed that ETV exhibited a smaller, yet significant, increase in creatinine compared with TAF (SMD = -0.55; 95% CI: -0.09 ~ -1.01) and TDF (SMD = -0.61; 95% CI: -0.15 ~ -1.06). Meanwhile, a comparison of the influence of TAF and TDF on the degree of creatinine rise was not statistically significant (SMD = 0.06; 95%CI: -0.03 ~ 0.15) (Fig. 4b).

**Fig. 5:**
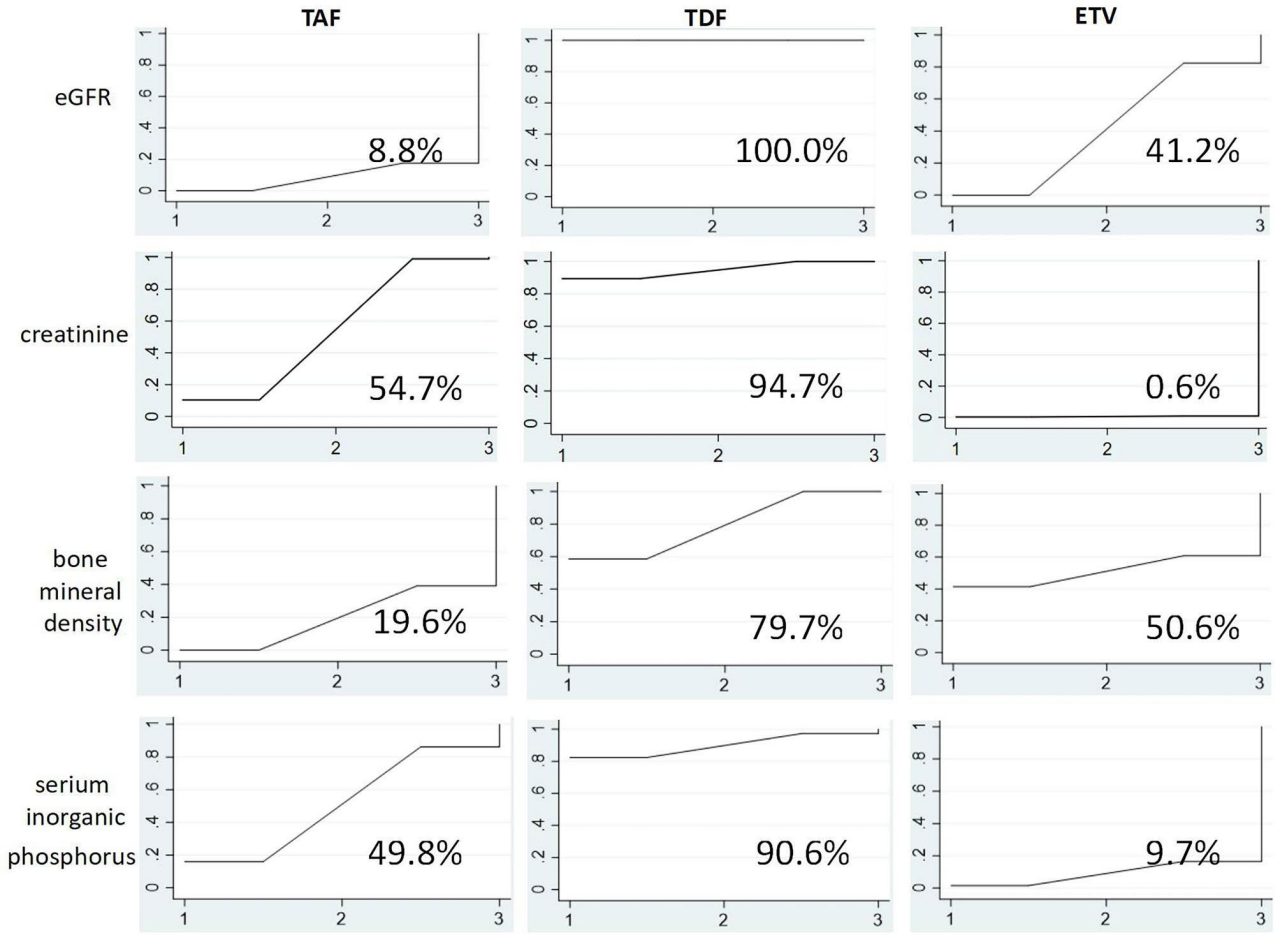
SUCRA diagram of side effect. The figure shows the probability of the effects of three drugs on eGFR, creatinine, bone mineral density, and blood phosphorus before and after medication. According to the level of area under the curve (SUCRA), the larger the area, the greater the index change value

As shown in Fig. 5, TAF exhibited the lowest eGFR reduction probability (SUCRA 8.8%), followed by ETV (SUCRA 41.2%). In contrast, TDF exhibited the highest eGFR reduction probability (SUCRA 100.0%). Regarding creatinine, ETV was least likely to increase creatinine (SUCRA 0.6%), while TAF (SUCRA 54.7%) and TDF (SUCRA 94.7%) were more likely to increase creatinine, respectively. Taken together, these data indicate that TDF adversely affected kidney tissue to a greater extent compared with TAF or ETV.

#### Bone events

To examine the effects of TAF, TDF, and ETV on bone mineral density, differences in lumbar bone mineral density were analyzed before and after treatment among 1,419 subjects in four studies. Differences in blood phosphorus before and after treatment in 926 subjects in eight studies were also analyzed.

As shown in Fig. 4c, TAF significantly reduced bone mineral density less than TDF (SMD = -0.02; 95%CI: -0.01 ~ -0.02). In contrast, there was no significant difference in bone mineral density observed between ETV and TDF, or between ETV and TAF (SMD = -0.01; 95%CI: -0.08 ~ 0.07 and SMD = 0.01; 95%CI: -0.07 ~ 0.09, respectively). Therefore, TAF exhibited a smaller effect on bone mineral density than TDF, and ETV was not comparable to TAF and TDF. Meanwhile, there were no statistically significant differences in the levels of blood phosphorus among the three drugs (Fig. 4d).

As shown in Fig. 5, TAF exhibited the lowest probability of decreasing BMD (SUCRA 19.6%), followed by ETV (SUCRA 50.6%). Conversely, TDF had the highest probability of decreasing BMD (SUCRA 79.7%). Regarding blood phosphorus, ETV had the lowest probability of reducing blood phosphorus (SUCRA 9.7%), followed by TAF (SUCRA 49.8%). TDF had the highest probability of reducing blood phosphorus (SUCRA 90.6%). Based on these two sets of data, TDF adversely affected bone tissue to a greater extent compared with TAF or ETV.

### Bias analysis

**Fig. 6:**
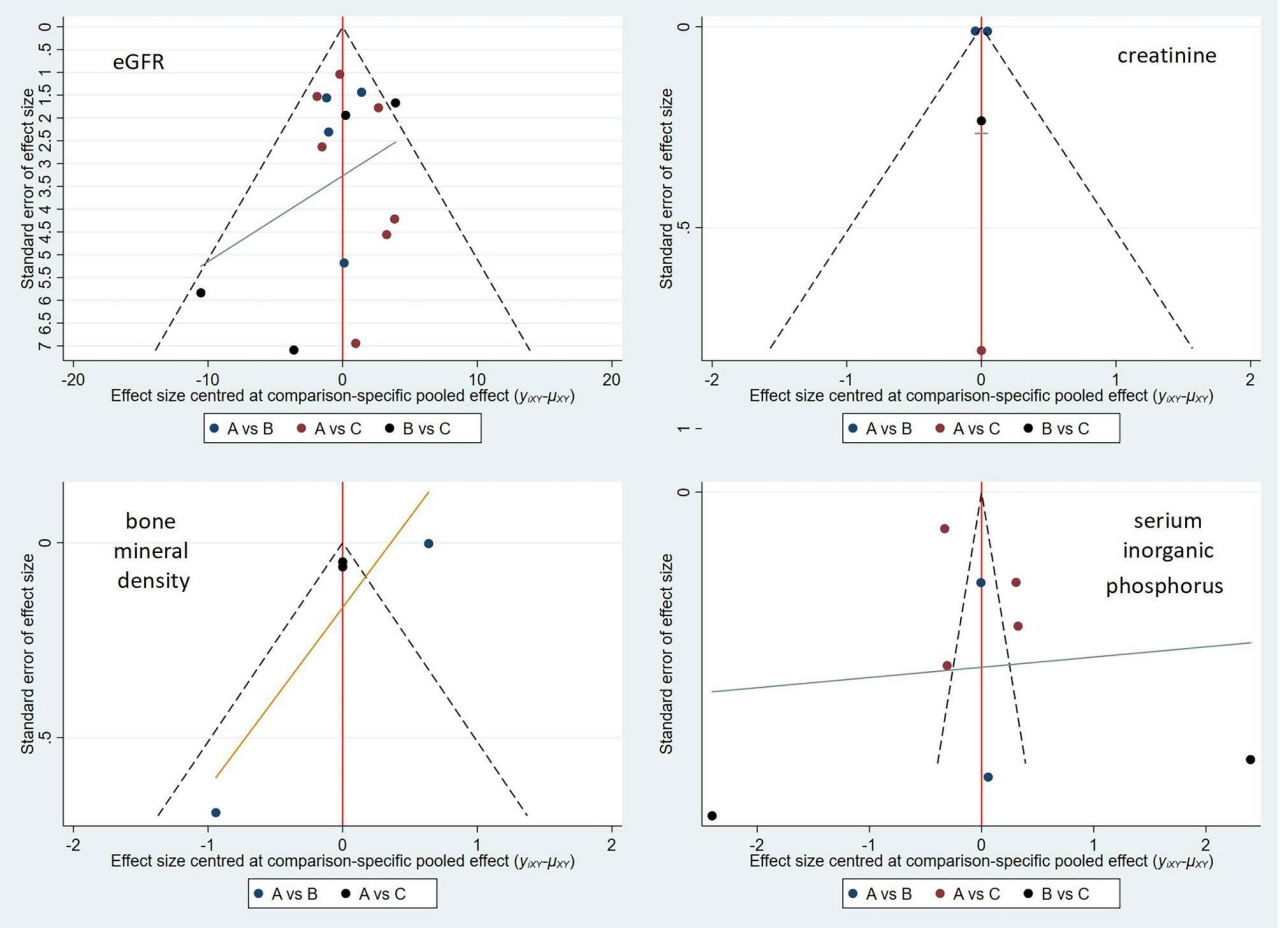
Comparison-adjusted funnel plots of each side effect to indicate bias

Stata 15 software was used to generate the funnel plot shown in Fig. 6. Each point in the triangle in the figure is roughly symmetrical with the central axis, indicating controllable publication bias.

### Subgroup analysis of duration of medication

**Fig. 7:**
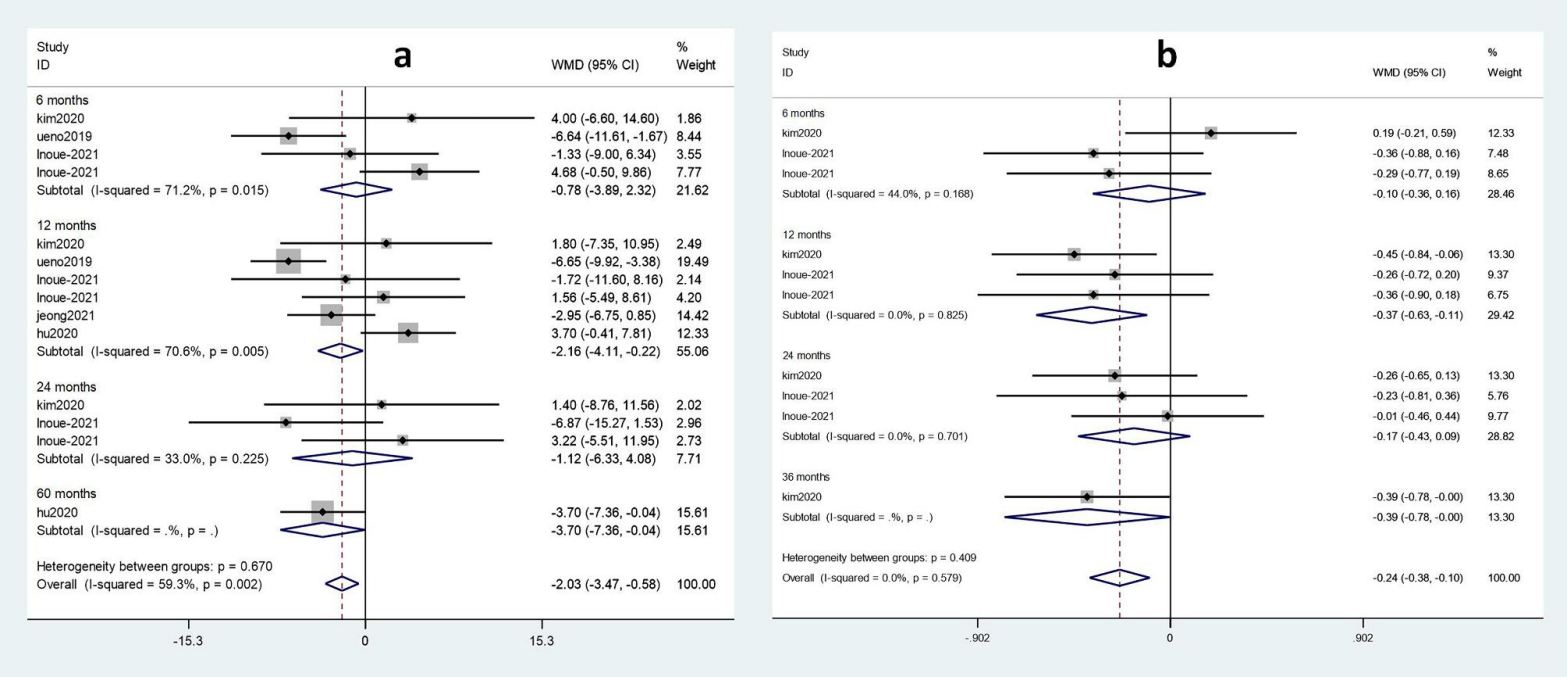
Forest plot of TDF and ETV subgroup analysis of eGFR and blood phosphorus according to duration of treatment. **a**) The impact of TDF on eGFR compared with ETV. **b**) The effect of TDF on blood phosphorus compared with ETV

The effects of TDF and ETV on eGFR and blood phosphorus were subjected to a subgroup analysis according to the duration of drug administration. Renal function and bone tissue damage in patients treated with TDF and ETV for various durations of treatment are shown in Fig. 7.

No statistically significant effect of the two drugs on eGFR occurred at 6 months (weighted mean difference (WMD) = -0.78; 95%CI: -3.89 ~ 2.32) or at 24 months (WMD = -1.12; 95%CI: -6.33 ~ 4.08) (Fig. 7a). However, a comparison of drug effects after 12 months (WMD = -2.16; 95%CI: -4.11 ~ -0.22) and after 60 months (WMD = -3.70; 95%CI: -7.36 ~ -0.04) demonstrates that TDF exhibited a greater adverse effect on eGFR than ETV at 60 months.

As shown in Fig. 7b, there was no statistically significant effect on blood phosphorus observed for the two drugs after 6 months (WMD = -0.10; 95%CI: -0.30 ~ 0.16) or after 24 months (WMD = -0.17; 95%CI: -0.43 ~ 0.09). In contrast, when the duration of medication was 12 months (WMD = -0.37; 95%CI: -0.63 ~ -0.11) or 36 months (WMD = -0.39; 95%CI: -0.78 ~ 0.00), a statistically significant effect on blood phosphorus was observed. Thus, compared with ETV, TDF reduced blood phosphorus levels more significantly at 36 months than at 12 months.

## Discussion

At present, there is international consensus that TAF, TDF, and ETV are first-line treatments for chronic hepatitis B [3, 26]. However, potential side effects due to long-term use of these drugs, especially in regard to kidney function and bone, remain to be determined. The aim of this meta-analysis was to explore side effects associated with long-term use of the common nucleoside analogues, TAF, TDF, and ETV, particularly in relation to renal function and bone, in chronic hepatitis B patients.

Many studies have shown that nucleoside analogues induce nephrotoxicity. Briefly, nucleoside analogues are transported to mitochondria-rich proximal renal tubular cells by transporters present in basal cell membranes [16]. However, when the intracellular concentration of nucleoside analogues exceeds a threshold, DNA polymerase activity is inhibited, thereby inhibiting mitochondrial replication. This ultimately leads to mitochondrial dysfunction, respiratory chain damage, lactic acid accumulation, and production of reactive oxygen species. Since mitochondria provide the energy necessary to reabsorb electrolytes and small molecules filtered by the glomeruli [27, 28], active uptake of nucleoside analogues may lead to intracellular accumulation of proximal tubule drugs in a dose-dependent manner, resulting in persistent renal tubular injury and a decline in eGFR [11, 26, 29].

Previous studies have shown that TAF exhibits higher plasma stability than TDF, primarily because both TAF and TDF are precursors of tenofovir (TFV). TAF and TDF are metabolized to TFV, then intracellular metabolization of TFV to tenofovir diphosphate provides an effective product. Higher plasma concentrations of TFV have been associated with greater damage to renal tubules. Moreover, higher intracellular concentrations of tenofovir diphosphate in cells induce a stronger antiviral effect. The intracellular concentration of tenofovir diphosphate derived from TAF, which represents a new nucleoside analogue, is about four times higher than that of TDF, while the plasma concentration is only 10% of TDF. As a result, TAF can achieve the same, or better efficacy, at a dose of approximately 10% of TDF, while also providing better renal and bone safety [30–33].

We included studies related to renal function indices, creatinine and eGFR, for our network meta-analysis. After selecting sixteen studies involving 4278 adults with chronic hepatitis B who were treated with ETV, TDF, or TAF, we observed that TDF was associated with a greater adverse effect on renal function than TAF or ETV. These observations were consistent with a pharmacokinetic study conducted on TAF and TDF. In this study, it was also observed that the effect of ETV on creatinine was less than that of TAF, and the difference was statistically significant (SMD = -0.55, 95%CI: -0.09 ~ -1.01). This result suggests that TAF has a greater side effect on renal function than ETV. It also provides a direction for us to further explore the effect of ETV and TAF on renal function. However, due to limited data, eGFR was not statistically significant in comparison between the two drugs, so more data and further statistical analysis are still needed.

Current studies have demonstrated that the damaging effect of nucleoside analogues on bone tissue is caused by renal injury. Hypophosphatemia secondary to proximal tubule injury due to nucleoside analogues may lead to insufficient bone matrix mineralization and the development of osteomalacia. In addition, dysfunction of proximal tubules may decrease hydroxylation of vitamin D which primarily occurs in the proximal tubules [12, 16, 28]. To examine skeletal effects, we included studies related to bone mineral density and blood phosphorus in our meta-analysis. Differences in lumbar bone mineral density before and after treatment in 1419 subjects from four studies, and differences in blood phosphorus before and after treatment in 926 subjects from eight studies, were analyzed. We observed that TDF adversely affected bone tissue to a greater extent compared with TAF or ETV. However, when TAF and ETV were compared, the two indices showed different results, and no unified conclusion could be made. These insights also suggest that future studies of nucleoside analogues should investigate whether the concentration of plasma exposure is reduced and the concentration of intracellular antiviral active substances is increased.

When subgroup analyses of the studies that used ETV and TDF were conducted according to duration of treatment, no significant difference in renal function or bone tissue was observed for either of the two drugs after 6 months of treatment. However, greater adverse effects on renal function were associated with TDF than ETV at 60 months (WMD = -3.70; 95%CI: -7.36 ~ -0.04) versus after 12 months (WMD = -2.16; 95%CI: -4.11 ~ -0.22). Greater reduction of blood phosphorus was also associated with TDF treatment at 36 months (WMD = -0.39, 95%CI: -0.78 ~ 0.00) than at 12 months (WMD = -0.37; 95%CI: -0.63 ~ -0.11). Therefore, a longer duration of TDF treatment resulted in greater adverse effects on renal and bone tissues than with ETV. However, neither renal function nor bone tissue exhibited any significant differences after 24 months of treatment. Since TAF has only been available for a short period of time, there are few relevant studies. Consequently, subgroup analyses could not be conducted. Overall, the difference in side effects between ETV and TDF was independent of treatment duration. Furthermore, due to the lack of relevant data, our conclusions may be biased. Therefore, renal and bone side effects due to treatment with nucleosides for longer than 36 months remain to be further investigated.

TAF, TDF, and ETV are all first-line drugs that are recommended for the treatment of chronic hepatitis B due to their antiviral effect. To date, low rates of drug resistance and high safety efficacy have been observed for these drugs. For example, long-term application of ETV has a resistance rate of 1%, while TDF and TAF exhibit no resistance [4]. The virological response of the three drugs has also reached greater than 90%, with the virological response rates of TDF and TAF being slightly higher than that of ETV [4]. Although the effect of TAF on kidney and bone damage is minor, the price of the newly available TAF drug is higher than TDF and ETV in China. This may impact whether it is selected as a treatment. Moreover, given the limited number of relevant studies included in the present study, the indicators examined may not represent a sufficiently comprehensive readout of bone injury, and our conclusions could be limited. In the future, relevant clinical studies are needed to explore the effects of different drugs on renal function and bone tissue in patients with chronic hepatitis B, especially regarding adverse effects on bone tissue. This would allow a more extensive meta-analysis to be conducted and to provide comprehensive conclusions and better guidance regarding clinical treatment.

## Conclusion

By conducting a network meta-analysis of studies which administered nucleoside analogues and evaluated long-term effects on renal function and bone tissue in chronic hepatitis B patients, we conclude that TDF exhibits stronger side effects on renal function and bone tissue than TAF and ETV. Moreover, in terms of creatinine, TAF exhibited a greater effect on creatinine increase than ETV. However, the degree of adverse reactions to bone tissue did not significantly differ between TAF and ETV. The difference in side effects between ETV and TDF was independent of treatment duration. Thus, further studies are needed. In particular, attention to renal function and bone-related indicators should be included.

### Electronic supplementary material

Below is the link to the electronic supplementary material.

Supplementary Material 1

Supplementary Material 2

## Data Availability

The datasets used and/or analyzed during the current study are available from the corresponding author on reasonable request.

## Abbreviations

CHB: Chronic hepatitis B
ETV: Entecavir
TAF: Tenofovir alafenamide
TDF: Tenofovir disoproxil fumarate
HBV: Hepatitis B virus
eGFR: Estimated glomerular filtration rate
BMD: Bone mineral density
SMD: Standardized mean difference
WMD: Weighted mean difference
95% CI: 95% Confidence interval
SUCRA: Surface under the cumulative ranking
