# Supplementary material for: Renal and bone side effects of long-term use of entecavir, tenofovir disoproxil fumarate, and tenofovir alafenamide fumarate in patients with Hepatitis B: a network meta-analysis
Source: BMC Gastroenterol. 2023 Nov 10;23:384. doi: 10.1186/s12876-023-03027-4 (PMC10638829; doi:10.1186/s12876-023-03027-4)
Supplement: Supplementary file 1 — Supplementary Material 1 [file 12876_2023_3027_MOESM1_ESM.docx]

**Identification of studies via databases and registers**

PubMed(n=464)
EMBASE(n=2870)
Cochrane Library(n=1628)

**Identification**

4962 articles were found

1564 duplicates

Excluded
Reasons for exclusion:
 1508 Not original investigation
 561 Not relevant outcomes
 1035 Not three targeted drugs
 294 Not human study

3398 Abstracts viewed

**Screening**

183 Full articles viewed

Excluded
Reasons for exclusion:
 9 Not RCT
 69 Not met the inclusion of intervention
 89 Not relevant outcomes

Include 16 trials from 16 articles, n=4078
 TAF vs TDF, 4 trials, n=1684
 TAF vs ETV ,6 trials, n=716
 TDF vs ETV, 5 trials , n=1315
 TAF vs ETV vs TDF, 1 trials , n=363

**Included**

*From:*  Page MJ, McKenzie JE, Bossuyt PM, Boutron I, Hoffmann TC, Mulrow CD, et al. The PRISMA 2020 statement: an updated guideline for reporting systematic reviews. BMJ 2021;372:n71. doi: 10.1136/bmj.n71

For more information, visit: <http://www.prisma-statement.org/>
